# Examine the parenting style effect on the academic achievement orientation of secondary school students: The moderating role of digital literacy

**DOI:** 10.3389/fpsyg.2022.1063682

**Published:** 2022-12-15

**Authors:** Mehdi Hassan, Asma Seemi Malik, Guoyuan Sang, Muhammad Rizwan, Iqra Mushtaque, Shaheryar Naveed

**Affiliations:** ^1^Institute of Teacher Education, Beijing Normal University, Beijing, China; ^2^Department of Sociology, Lahore College for Women University, Lahore, Pakistan; ^3^Institute of Teacher Education Beijing Normal University, Beijing, China; ^4^School of Education Sciences, Nanjing Normal University, Nanjing, China; ^5^Department of Psychology, Bahauddin Zakariya University, Multan, Sub-Campus Layyah, Pakistan; ^6^Department of Public Administration, Fatima Jinnah, Women University, Rawalpindi, Pakistan

**Keywords:** education, parenting style, digital literacy, self-regulated e-learning, computer self-efficacy

## Abstract

The purpose of the study was to determine the association between parenting styles (authoritative and permissive) and students’ self-efficacy (LSE), self-regulatory learning (SRL), and academic accomplishment orientation of secondary school students in Punjab, Pakistan. The study also investigated the effect of digital learning as a moderating variable in the relationship between SRL and academic achievement oriented, as well as between learner self-efficacy (LSE) and academic achievement among secondary school students. The study was conducted with (*N* = 720) secondary school students of Punjab Pakistan. In the current research cross sectional design was used, and multistage sampling was used to draw a sample from the population. The results from the study, it is found that the authoritative parenting style has a weak association with LSE and a strong association with SRL. Permissive parenting styles have low associations with SRL and have a high association with LSE as compared to authoritarian parenting. Furthermore, when compared to students from permissive parenting, secondary students from authoritarian parenting have higher SRL and a higher academic achievement orientation. Results revealed that that digital literacy significantly moderate with LSE to influence the academic achievement orientation, while digital literacy significantly interacts with SRL to highly influence the academic achievement orientation of secondary school students.

## Introduction

Parenting is a sunshade word that goes on to talk about all forms of parental involvement with students and the kind of relationship, which exists between them (Mushtaque et al., 2021a). According to Carlo et al. (2017) believes that parents’ responsibility is to raise their students and become responsible citizens. Parenting approaches in the lives of students inside the home establish numerous social environments (Davis et al., 2015). Parenting styles vary culturally. In Asian countries like Pakistan, parenting styles refer to how parents control their children’s lives. Parental behaviors and attitudes affect children’s early and future lives. Children need solid parent–child connections to behave consistently, be self-sufficient, gain social skills, and become independent. This is connected to parental attitudes and behaviors, or parenting approaches. Authoritative, authoritarianism, permissiveness, apathy, and overprotection are common parenting styles (Checa et al., 2019). Children are overseen by Authoritative parents, but their urgent needs are considered. Authoritative environment encourages youngsters to trust themselves within fair bounds and develops healthy sovereignty (Dakers and Guse, 2020). Children raised in a democratic family can be confident, self-sufficient, creative, and healthy. It’s common in patriarchal societies (Kösterelioğlu, 2018). Extreme authoritarians focus on the child’s failure and mistakes rather than their own (Kezer and Turker, 2012; Scarcella et al., 2016). Permissive parents show warmth and care and do not set excessive expectations. According to them, the only way to love a child is to grant all their wants. Such parents would say, “Sure, you can stay out late.” Such parents do not want to disappoint their children, thus teens may make various decisions without telling them, believing they will not agree. This can lead to teen impulsivity and selfishness (Baumrind, 1991). There was an essential link between the permissive parenting style and students’ academic grades, the authoritarian parenting style, and the authoritative parenting style with the students’ average study grade. This study shows that parental influence plays an important role in students’ educational success (Rahimpour, 2015). The workload, psychological resources, and academic achievement of primary school students were examined by the researchers. Academic self-efficacy, self-regulated learning, and academic performance are all influenced by parental support for academic autonomy. Parental control correlated negatively with academic self-efficacy, self-regulated learning, and success. Parental involvement in homework is associated with children’s psychosocial and cognitive development. Academic achievement is connected to parental homework involvement (Grijalva-Quiñonez et al., 2020; Mushtaque et al., 2021b).

Academic self-efficacy is defined as a student’s confidence in their capacity to successfully perform academic assignments (Alzig, 2009). Academic confidence gained *via* academic success is referred to as self-efficacy. Self-efficacy is defined intrapersonal as a student’s belief in his or her ability to succeed in school (Pampaka et al., 2018). According to Fan and Williams, self-efficacy influences students’ effort and persistence (Fan and Williams, 2009). Students with high levels of self-efficacy are more likely to put forth the effort necessary to overcome academic obstacles. Students’ ability to self-regulate their learning is critical to their success in higher education. Teachers and students may benefit from learning analytics to better understand how students learn (Mushtaque et al., 2021c). Learning analytics capabilities are expected to aid students in planning and organizing their learning processes, as well as self-assessment, adaptive recommendations, and analysis of their learning activities (Schumacher and Ifenthaler, 2018; Aqeel et al., 2022). Academic success involves self-directed learning (SRL). It is vital to use SRL in technology-based learning (Winters et al., 2008). According to Valentn et al., the interaction between SRL techniques and technology may be haphazard (Valentín et al., 2013).

Many teachers presume today’s students are digitally literate because they are engaged with technology and feel safe using it to perform chores. University students lack digital learning skills (Muresan and Gogu, 2013). Many college freshmen lack digital literacy. Students may use technology for social networking or entertainment instead of teaches (Prior et al., 2016). Not comprehending the ethical and social use of information (O’Sullivan and Dallas, 2010; Greene et al., 2014; Tang and Chaw, 2016). Digital learning also requires self-regulation skills. Students must be independent in digital learning. Self-regulated learners govern their learning. Self-regulated learning includes effort management. Digital literacy takes self-study (Greene et al., 2014). Online learning benefits from self-education and IT regulation (Ejubović and Puška, 2019). School and work require self-control and computer literacy (Scott, 1996). Other researchers say self-regulated learning’s impact on digital literacy is unknown (Broadbent, 2017). Promoting digital literacy in students requires more research because there is no one-size-fits-all strategy (Ting, 2015; Hassan et al., 2022). In digital learning situations, further study is needed on SRLS (Greene et al., 2014).

Based on the foregoing information, it appears that parents’ parenting style may impact students’ self-regulation, self-efficacy, and academic accomplishment. An authoritative and permissive parenting style may help students acquire self-regulation and achieve academically (Rizwan et al., 2021). A collaborative process in 2004–2005 resulted in Pakistan’s first-ever National Information and Communication Technology (NICT) Education Strategy Program. According to the NICT plan, technology has the potential to increase educational quality and accessibility, strengthen teacher preparation and aid student achievement (Batool, 2019). Digital literacy is the ability to use technology devices to access, manage, and use information. Teachers teach students how to use email, Google Classroom, Google Meet, and Frog-VLE interactive programs to help them become digital citizens. In the classroom, these media and technological elements are employed to engage students in interactive learning (Tohara and Al, 2021). While students can utilize technology devices, they struggle to get the information they need on technology platforms, indicating a lack of digital literacy. Higher levels of digital literacy may necessitate more technological and cognitive focus.

### Objectives of the study

Due to Pakistan’s emphasis on inclusive learning, students may be pushed to incorporate digital literacy abilities into their learning methodology. According to Rizwan et al. (2021) internal factors such as culture, ethnicity, family history, geography, and the schools they attend also contribute to students’ various learning styles. Parenting in Pakistan is also dissimilar from that of Western nations. Parenting styles vary by culture, and this has an effect on their children’s academic accomplishment. As a result, in Pakistani society, a study of how parenting styles influence their children’s academic achievement and how new technology adoption literacy affects their academic achievement is required.

In the current study, we examined the secondary students’ parenting styles; how a parenting style can influence the students’ technology-related self-regulated learning and their technology-related self-efficacy towards the academic achievement orientation, also examine the moderating role of digital literacy.

## Methodology

### Sample and participant

The quantitative research approach and deductive paradigm were used in the current study to collect data using a survey method. The participants were students at the higher secondary level studying in Punjab, a province of Pakistan. Parents of the same students also participated in the study. Multistage sampling was used to draw a sample from the population. Three divisions were selected out of nine divisions, one each from the southern, central, and northern Punjab. One developed and one under-developed district was chosen from each division, making a total of 6 (3*2 = 6). Four secondary schools were selected from each district, two girls and two boys (6 × 4 = 24), making a total of 24 schools. Ten students in the school (24*10 = 240) were selected, making 240 students from each division. Seven hundred and twenty was the sample targeted across 3 divisions. The study sample was comprised of 720 students, among whom 375 were male and 345 were female (Table 1).

**Table 1:** Descriptive statistics of demographics (*N* = 720).

Name	Frequency	Percent	*M* (*SD*)
**Gender**			
Male	375	52.1	
Female	345	47.9	
**Region**			
Northern Punjab	240	33.3	
Central Punjab	240	33.3	
Southern Punjab	240	33.3	
**Father occupation**			
Governmental	340	47.2	
Non-governmental	380	52.8	
**Mother occupation**			
Governmental	306	42.5	
Non-governmental	414	57.5	
**Father education**			
Uneducated	287	39.9	
Primary	159	22.1	
Secondary	242	33.6	
Master	32	4.4	
**Mother education**			
Uneducated	265	36.8	
Primary	222	30.8	
Secondary	72	10.0	
Bachelor	97	13.5	
Master	64	8.9	
**Use of computer**			2.1 (4.32)

The participants’ demographic information was described as follows: 375 male and 345 female students were selected from three regions of the Punjab province: Northern Punjab, Central Punjab, and Southern Punjab; from each region, 240 students were selected. The students whose father’s occupation was governmental were 340, and without governmental was 380. Those students whose mothers belonged to governmental occupations were 306, and those without governmental occupations were 414. The students whose father’s education was uneducated (287); primary (159); secondary (242) and master (32). Students with uneducated mother ratio were (265), primary (222), secondary (72) bachelor (97), and master (64). Students reveal that they used computer based application average 2 h per day.

### Instrument of the study

#### Parenting style scale

It is a self-reporting questionnaire. The scale has the three dimensions; authoritative, authoritarian and permissive parenting. This scale was developed by Situmorang and Salim (2021). It is a 7-point Likert scale (1–7); 1 = (never) and 7 = (always). The current study I examined the two domains authoritative parenting style and permission parenting style. The authoritative parenting style has the 10-itemns while permissive parenting style has the 10-items. The reliability of the scale was 0.90.

#### Self-regulation questionnaire

SRQ developed by Pichardo et al. (2018). It is a self-reported 4 items scale. It is a 5-point Likert scale (strongly disagree to strongly agree). The reliability of the scale was 0.89.

#### Learning self-efficacy scale

The researcher (Park, 2009) developed the questions to assess self-efficacy in e-learning. Two elements were utilized to determine it: confidence in locating knowledge in an e-learning system and the extent to which essential competencies were possessed. All constructs were assessed on a Likert scale of 1–7 on a seven-point scale. The reliability of the scale was 0.92.

#### Digital literacy scale

The participants’ digital nativity was assessed using Teo (2013) Digital Nativity Assessment Scale (DNAS). The Likert scale has four variables and 4 items. People on the scale are comfortable multitasking, rely on pictures for communication, and thrive on immediate satisfaction. The current study used a 5-item scale to assess participants’ multitasking ability. The reliability of the scale was 0.78.

#### Academic achievement orientation

The researcher (Baker and Sinkula, 1999) developed the questions to assess the academic achievement orientation. Five elements were utilized to determine it: ability to achieve goal, investment to learn, guarantee success, top priority and collective wisdom. All constructs were assessed on a Likert scale of 1–7 on a seven-point scale. The reliability of the scale was 0.86.

### Procedure

The institution’s head as well as the parents of eligible participating students were sent a consent letter describing the study and requesting students to participate. Upon receipt of written consent from parents, students were selected to participate in the survey. It was assured that their information would remain confidential and only be used for the study. Data were analyzed using both descriptive and inferential statistics. After collection of the data, the responses were quantified and the data was tabulated through the use of statistical packages for the social sciences (SPSS v. 25).

## Results

A temporal research utilizing structural equation modeling (SEM) was conducted to acquire the desired results. SEM has been demonstrated to be the most effective method for examining the relationship between a large number of indicators and criterion variables, as well as for estimating models that contain no measurement errors (Marcoulides et al., 2009). Additionally, AMOS does well when it comes to estimating formative measures and moderating correlations. Additionally, the AMOS algorithm generated graphs representing the latent concept’s hypothesized relationships. As a result, we used AMOS to identify the connection in our research.

### Reliability and convergent validity

Cronbach’s alpha, factor loadings, and average variance extracted (AVE) were used to examine the validity and reliability of the concept used in this study. We used CFA to evaluate the items’ validity and came to the conclusion that all exhibits should be trustworthy (Cronbach’s alpha >0.70). Each produced object’s factor loadings were found to be greater than 0.60. Finally, each produced value’s AVE surpasses the specified cutoff limit, i.e., AVE > 0.50. AVE > 0.50: The latent factor accounts for at least half of the variance between the items. CFA employed AMOS to determine whether the study’s findings indicated used to verify item-factor compatibility and prepare for factor The proposed model’s overall fit indices are valid (between 0.45 and 0.87), which is within the desired range based on the CFA results. Cronbach’s alphas were all positive. All of the relevant Cronbach’s alphas were over 0.80. Both CR and AVE delivered on their promises. The results of the reliability testing are shown in Table 2. As a result, it displays all of the test equipment. The construction was excellent. There appears to have been no cross-validation. Except for one item, loading is a challenge. As a result, it was not included in the construction.

**Table 2:** Factor loading and Cronbach’s alphas.

Scales	Indicator	Loading	Cronbach alpha	AVE	CR
	PS1	0.8133			
	PS2	0.8700			
	PS3	0.8661			
	PS4	0.8753			
Parenting style authoritative	PS5	0.8364	0.92	0.599	0.937
	PS6	0.7813			
	PS7	0.7434			
	PS8	0.6386			
	PS9	0.6024			
	PS10	0.6559			
	PS11	0.7015			
	PS12	0.7051			
	PS13	0.7965			
	PS14	0.7788			
Parenting style permissive	PS15	0.7826	0.92	0.522	0.916
	PS16	0.7623			
	PS17	0.6916			
	PS18	0.6880			
	PS19	0.6771			
	PS20	0.6285			
Self-efficacy	LSE1	0.8479	0.69	0.763	0.866
	LSE2	0.8986			
	SRL1	0.8415			
	SRL2	0.8275			
Self-regulation	SRL3	0.8361	0.85	0.691	0.900
	SRL4	0.8208			
	DL1	0.9687			
	DL2	0.9338			
Digital learning	DL3	0.9157	0.95	0.874	0.965
	DL4	0.9203			
	AAO1	0.8437			
	AAO2	0.8264			
	AAO3	0.8634			
Academic achievement orientation	AAO4	0.7737	0.88	0.685	0.916
	AAO5	0.8294			

### Discriminant validity

In addition to convergent validity, we examined the discriminant validity of our suggested construction. For each of our constructs, we looked at the AVE. The AVE of each construct outperforms the correlations between constructs. There were no discriminant validity difficulties with the AVE > inter-construct correlations. The inter-construct correlation matrix (Table 3) indicates that each construct’s AVE (bold and diagonal) is greater than the variable correlation. We determined that all values were above the acceptable level for cross-loading between constructs, indicating that there were no cross-loading concerns. Table 4 shows the cross-loading of each built component. There are no significant cross-loading difficulties found, resulting in a “high level of discriminant validity.”

**Table 3:** Construct correlation.

Construct	PS-A	LSE	SRL	AAO	DL	PS-P
PS-A	**0.774**					
LSE	0.296	**0.873**				
SRL	0.580	0.257	**0.831**			
AAO	0.598	0.230	0.681	**0.828**		
DL	0.148	0.003	0.091	0.078	**0.935**	
PS-P	0.576	0.389	0.441	0.407	0.089	**0.722**

PS-A, parenting style authoritative; LSE, learners self-efficacy; SRL, self-regulatory learning; AAO, academic achievement orientation; DL, digital literacy; PS-P, parenting style permissive. Table shows the discriminant validity acceptance values and shows that all of them are within an acceptable range (Scale Authoritative and Permissive parenting style has the Scale Validity 0.77 and 0.72). Similarly, the scores for learner self-efficacy (0.87), self-regulatory learning (0.83), and academic achievement oriented (0.82), and digital literacy in our study had validity (0.93). The researchers tested the factor loadings of each item of the all study variables and discovered that all values were between 0.500-0.900.

**Table 4:** Cross loadings.

Indicator	PS-A	LSE	SRL	AAO	DL	PS-P
PS1	0.8133	0.2618	0.4833	0.4866	0.1315	0.3403
PS2	0.8700	0.2713	0.5277	0.5439	0.1355	0.4305
PS3	0.8661	0.1952	0.4609	0.5134	0.1215	0.4082
PS4	0.8753	0.2250	0.4603	0.5231	0.1735	0.3829
PS5	0.8364	0.2495	0.4414	0.4682	0.1392	0.3457
PS6	0.7813	0.2247	0.4253	0.4553	0.1241	0.3368
PS7	0.7434	0.2007	0.3991	0.4040	0.1043	0.3934
PS8	0.6386	0.2095	0.4076	0.3675	0.1093	0.5759
PS9	0.6024	0.2220	0.4134	0.3861	0.0313	0.6368
PS10	0.6559	0.2189	0.4405	0.4455	0.0658	0.6413
PS11	0.6215	0.2536	0.4040	0.3961	0.0954	0.7015
PS12	0.5346	0.2361	0.3798	0.3018	0.0964	0.7051
PS13	0.5392	0.2212	0.4038	0.3745	0.1109	0.7965
PS14	0.5627	0.1847	0.4189	0.3744	0.0897	0.7788
PS15	0.2331	0.3318	0.2226	0.1877	0.0255	0.7826
PS16	0.2217	0.3102	0.1807	0.2098	0.0445	0.7623
PS17	0.2456	0.2712	0.1873	0.1850	0.0263	0.6916
PS18	0.2227	0.3110	0.1942	0.1871	0.0366	0.6880
PS19	0.2089	0.2798	0.1954	0.1751	0.0170	0.6771
PS20	0.2818	0.2555	0.2555	0.2460	0.0128	0.6285
LSE1	0.2357	0.8479	0.2004	0.1900	0.0489	0.2988
LSE2	0.2799	0.8986	0.2457	0.2122	0.0342	0.3753
SRL1	0.4798	0.2098	0.8415	0.5462	0.1189	0.3604
SRL2	0.4881	0.1995	0.8275	0.5428	0.0698	0.3719
SRL3	0.4999	0.2311	0.8361	0.5432	0.0760	0.3895
SRL4	0.4632	0.2145	0.8208	0.6315	0.0403	0.3453
DL1	0.1305	0.0028	0.0790	0.0747	0.9687	0.0816
DL2	0.1648	0.0096	0.1046	0.0940	0.9338	0.0782
DL3	0.1274	0.0459	0.0720	0.0312	0.9157	0.0805
DL4	0.1337	0.0240	0.0854	0.0928	0.9203	0.0927
AAO1	0.4793	0.1986	0.5727	0.8437	0.0708	0.3193
AAO2	0.4265	0.2166	0.5392	0.8264	0.0088	0.3115
AAO3	0.5209	0.1635	0.5674	0.8634	0.0119	0.3625
AAO4	0.5518	0.1837	0.5542	0.7737	0.1118	0.3346
AAO5	0.4993	0.1930	0.5845	0.8294	0.1181	0.3577

PS-A, parenting style authoritative; LSE, learners self-efficacy; SRL, self-regulatory learning; AAO, academic achievement orientation; DL, digital literacy; PS-P, parenting style permissive.

### Measurement and structural model

To determine the models’ fit to the data, a number of fit statistics were examined (Table 5). All essential path values, correlations for each variable and overall model fit statistics were calculated by AMOS. Model-data fit is assessed using fit indices. The overall chi square value, as well as degrees of freedom (df), the chi square to degrees of freedom ratio, comparative fit index (CFI), normed fit index (NFI), and root mean square error of approximation (RMSEA) with a 90% confidence interval, were recommended by Lachenbruch and Cohen (1989). A CFI is a metric that assesses how well two people fit together. Null goodness of fit (all variables are non-correlative) and saturated goodness of fit (a model with 0 degrees of freedom that exactly reproduces the original covariance matrix) are the two types of goodness of fit (Schumacker and Lomax, 2004). The CFI scale runs from 0 to 1.0, with a score of 0.90 indicating satisfactory fit. Similarly, proposed a cut-off value of 0.95 or above. A CFI of 0.90 indicates that the model outperforms the null model based on the same sample data by 90%. From 0 to 1, the normalized fit index (NFI) scales. The original covariance matrix is accurately reproduced by a saturated model or a model with 0 degrees of freedom (Schumacker and Lomax, 2004). The NFI, like the CFI, has values ranging from 0 to 1, with a value of 0.90 indicating adequate fit (Schumacker and Lomax, 2004). The RMSEA evaluates model complexity in addition to model fit and is less dependent on sample characteristics than the chi square. Model fit is defined as being less than 0.06, and moderate model fit as being between 0.06 and 0.10 Over 0.10 indicates a poor match (Glaser, 2008). The RMSEA 90 percent confidence interval should be assessed and reported (Glaser, 2008).

**Table 5:** Model fit indices of measurement and structural model.

Indices to measure fitness	Confirmatory (CFA)	Model (proposed)	Cutoff value
CMIN/df	3.954	4.014	
CMIN	2068.114	2127.670	
Degree of freedom	523	530	Less than 5.0 Hu and Bentler (1999)
GFI	0.862	0.858	Above 0.90 Bélanger and Carter (2009)
AGFI	0.834	0.831	Above 0.80 Bélanger and Carter (2009)
CFI	0.927	0.925	Above 0.90 Bélanger and Carter (2009)
RMSEA	0.063	0.063	Below 0.8 Bélanger and Carter (2009)
NFI	0.905	0.903	Above 0.90 Bélanger and Carter (2009)
TLI	0.917	0.916	Above 0.90 Bélanger and Carter (2009)
IFI	0.928	0.925	Above 0.90 Bélanger and Carter (2009)

### Testing of hypothesis

1. Authoritative parenting style has a significant association with learners self-efficacy (LSE) among Pakistani secondary school students.
2. Authoritative parenting style has a significant association with self-regulatory learning (SRL) among Pakistani secondary school students.
3. Permissive parenting style has a significant association with LSE among Pakistani secondary school students.
4. Permissive parenting style has a significant association with SRL among Pakistani secondary school students.
5. SRL has a significant association with academic achievement orientation among Pakistani secondary school students.
6. LSE has a significant association with academic achievement orientation among Pakistani secondary school students.
7. Digital literacy would moderate the relationship between SRL and academic achievement orientation among Pakistani secondary school students.
8. Digital literacy would moderate the relationship between self-efficacy and academic achievement orientation among Pakistani secondary school students.

The alternative model fit summary indicates that the model is the fit for all the alternatives. When alternative model 1 (1-2-1) was adopted, the authoritative parenting style was checked with the self-efficacy and SRL and outcome variable academic achievement orientation. Similarly, permissive parenting style was assessed using the same model (1-2-1). In alternative model 3, all the predicator’s variables were checked on the criterion variable (Table 6).

**Table 6:** Alternate models fit summary.

	Models	CMIN	Df	CMIN/df	Non-centrality		Relative		Absolute	
					CFI	RMSEA	TLI	NFI	GFI	AGFI
1	Independent	21852.465	595							
2	Measurement	2068.114	523	3.954	0.927	0.063	0.917	0.905	0.862	0.834
3	Hypothesized	2127.670	530	4.014	0.925	0.063	0.916	0.903	0.858	0.831
4	Alternate 1 (P1)	2438.296	532	4.583	0.910	0.069	0.900	0.888	0.836	0.806
5	Alternate 2 (P2)	2197.572	531	4.139	0.922	0.065	0.912	0.899	0.854	0.827
6	Alternate 3 (all)	2089.132	528	3.957	0.927	0.063	0.917	0.904	0.860	0.833

**Figure 1:**
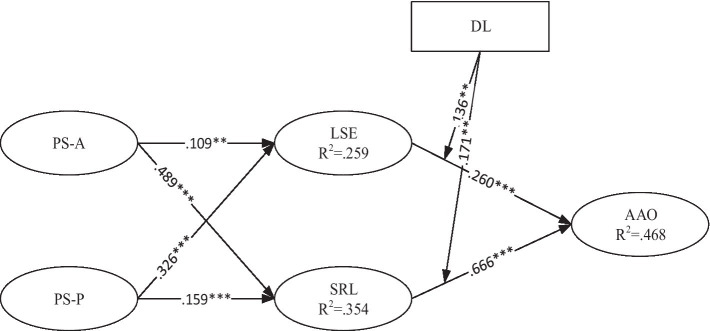
Results of the model test with moderating effect. **p < 0.01, ***p < 0.001.

The results from the structural analysis supported the hypothesis (Figure 1; Table 7). Results indicate that the authoritative parenting style has a significant low association with LSE (*H*1: *β* = 0.109, *p* < 0.001), while on the scale of SRL; the authoritative parenting style has a moderate association (*H*2: *β* = 0.489, *p* < 0.001). On the scale of the permissive parenting style, self-efficacy has a better association as compared to the authoritative parenting style (*H*3: *β* = 0.326, *p* = 0.001), and on SRL (*H*4: *β* = 0.159, *p* < 0.001). The results revealed that a permissive parenting style has a weak association on the SRL scale. The relationship between LSE and academic achievement orientation was also assessed by using path analysis. The results revealed that LSE and academic achievement orientation have a significant positive association and support the hypothesis (*H*5: *β* = 0.260, *p* < 0.001). Secondary school students’ self-regulated learning was also assessed with the academic achievement orientation, and the results were significant (*H*6: *β* = 0.666, *p* < 0.001).

**Table 7:** Summary of hypothesis testing.

	Hypothesizes	Significance	Finding
*H*1	PS-A + →LSE+	0.109***	Supported
*H*2	PS-A + →SRL+	0.489***	Supported
*H*3	PS-P + →LSE+	0.326***	Supported
*H*4	PS-P + →SRL+	0.159***	Supported
*H*5	LSE + →AAO+	0.260***	Supported
*H*6	SRL + →AAO+	0.666***	Supported
*H*7	LSE* DL →AAO	0.136**	Supported
*H*8	SRL* DL →AAO	0.171***	Supported

***p ≤ 0.001, **p ≤ 0.01, *p ≤ 0.05. PS-A, parenting style authoritative; LSE, learners self-efficacy; SRL, self-regulatory learning; AAO, academic achievement orientation; DL, digital literacy; PS-P, parenting style permissive.

**Figure 2:**
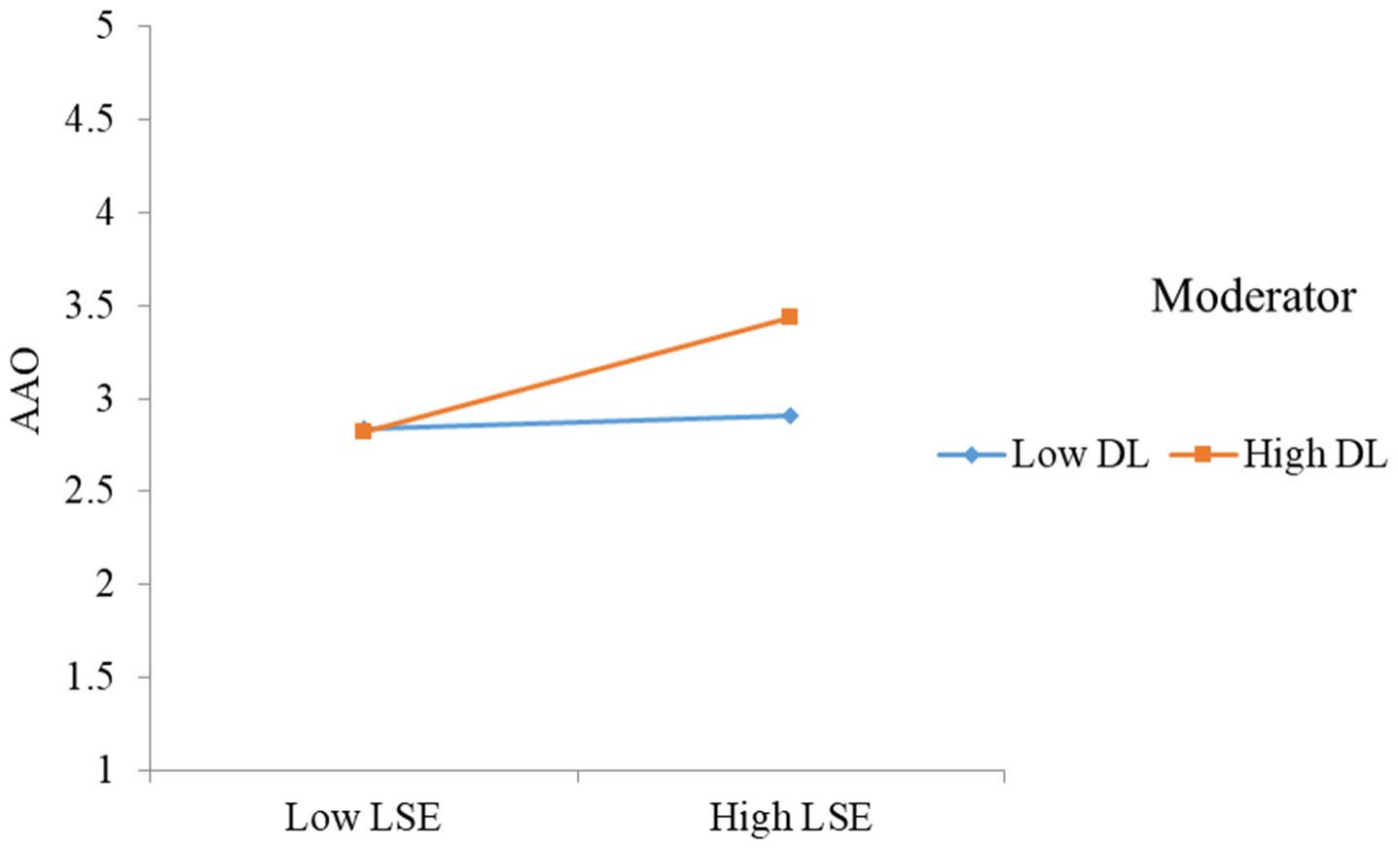
Moderating effect of digital literacy.

**Figure 3:**
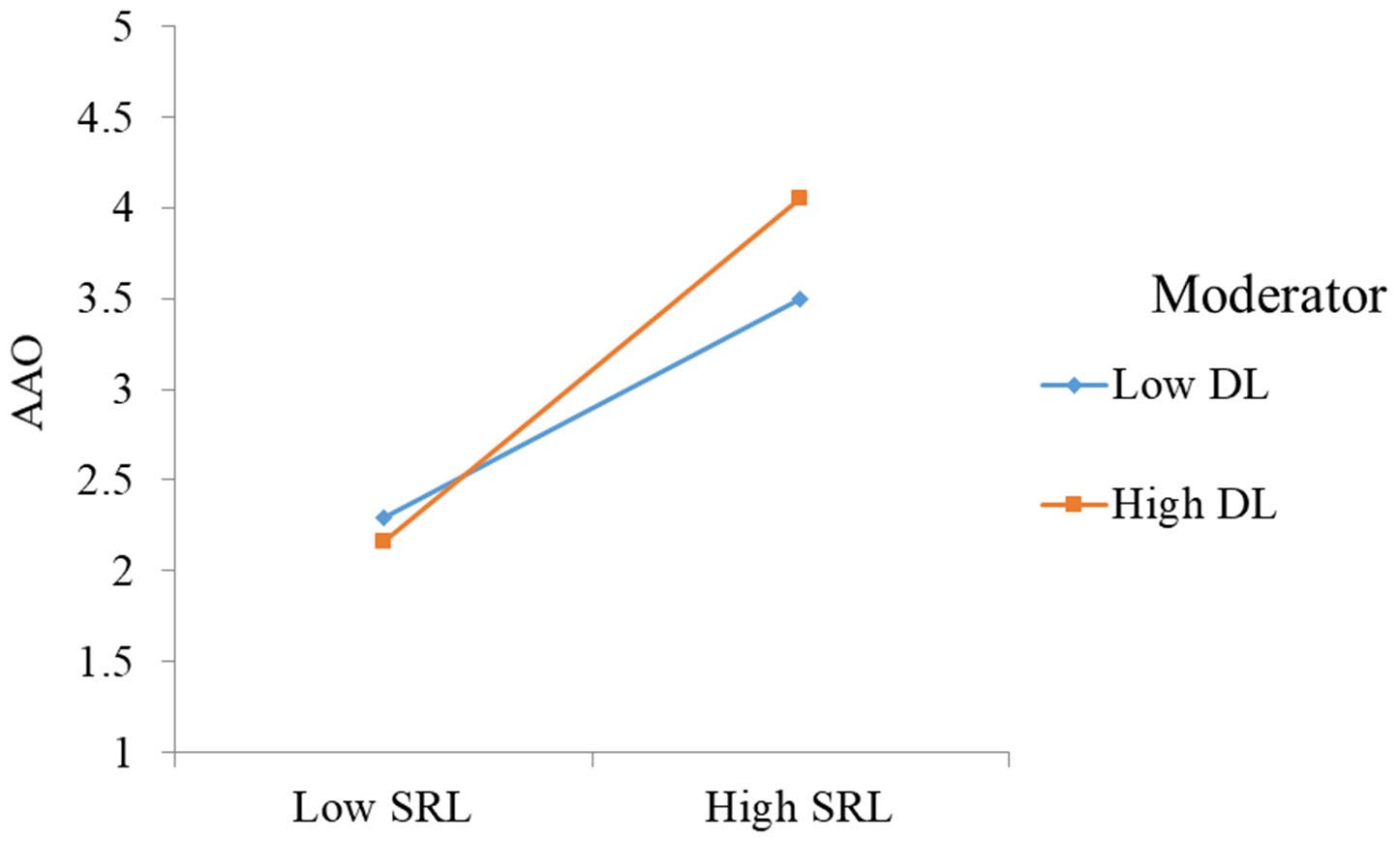
Moderating effect of digital literacy.

In this research one moderator was discussed is digital literacy, detail of the moderator effects are given in Figures 2, 3. The moderating effects of digital literacy strengthen the relationship of SRL, self-efficacy on academic achievement orientation. In the current study we used the approach to examined the moderating effects in structural equation model. According to this approach, a means-centered indicator should be used before creating the link. So, to test the moderation effect of digital literacy, a connection has to be created between self-efficacy, SRL, and academic achievement orientation. In keeping with the hypotheses of *H*7 and *H*8, we found that digital literacy significantly interacts with LSE to influence the academic achievement orientation (*H*7: *β* = 0.136, *p* < 0.01), while digital literacy significantly interacts with SRL to highly influence the academic achievement orientation (*H*8: *β* = 0.171, *p* < 0.001). The plot presented in the Figure 3 in which digital literacy strengthen the relationship between LSE and (AAO) and Figure 1 depicts that digital literacy (DL) strengthen the relationship between SRL and academic achievement orientation (AAO).

## Discussion

Parenting style influence on student SRL and academic achievement may differ. According to Carlo et al. (2017) children of authoritarian parents have self-competence beliefs when faced with academic challenges and obstacles. While the permissive parenting style has multiple positive outcomes in general, but in some researches have looked into the beneficial effects of the authoritarian parenting style (Checa et al., 2019). As in the current study, parenting style was evaluated in the context of Pakistani culture and evaluates the academic achievement orientation of secondary school students.

As the current study results indicate that the authoritative parenting style has a significant low association with LSE, while on the scale of SRL; the authoritative parenting style has a moderate association (Table 7; Figure 1). Results indicated that students with authoritative parenting style has the positive but low association with LSE while has the high association with SRL. One of the key determinants of children’s academic self-efficacy is their lifelong assessment of parenting styles. The consequences of parenting practices can be explained in terms of students’ experiences with mastery, vicarious, and social persuasion. According to Rahimpour (2015), parenting styles have a significant impact on all stages of a person’s life, from preschool to college. According to Rivers et al. (2012), parents impact the development of self-efficacy by providing observational models that might guide adolescents’ shifts in self-perceptions. Additionally, the studies indicate that parents’ affirmation of their children’s skills results in poor self-esteem and a great capacity for overcoming hurdles. Parenting style is mostly a situational factor affecting academic accomplishment. On the other hand, academic self-efficacy may function as a positive relationship between the effects of parenting styles on adolescent academic attainment (Vaculíková, 2018). Authoritarian and permissive parenting styles were also prevalent in Pakistani society. The findings support previous study, particularly for school children (Jamil and Mahmud, 2019). Pakistani parents appear to enforce norms, guidelines, and boundaries on their children while remaining open to listening to and conversing with them. Permissive parenting is less common in the Middle East and East, whereas these two parenting styles are more prominent (Farooq and Asim, 2020). This parenting style (very authoritative, lowly tolerant) supports children’s development. According to early parenting research, this mix of parenting approaches has favorable psychological, intellectual, social, and behavioral outcomes for children. Moreover, the findings suggest that parenting style (authoritative) has an impact on secondary school students’ academic achievement. Parenting approaches (authoritative), academic self-efficacy, and accomplishment were among the student findings. The study showed significant differences in the schooling of the mothers of the students. In terms of responsiveness and control, the study indicated that parenting styles vary little (Rizwan et al., 2021).

It’s also worth noting some of the study’s most significant findings. The first is on the authoritarian style’s significant benefits for self-regulated learning in schoolchildren. Through cross-cultural research, inconsistent findings about the effect of authoritarian parenting on children’s development were uncovered. According to certain experts, notably in the Middle East and Eastern countries, authoritarian parenting has a beneficial effect on children’s outcomes (Keshavarz and Mounts, 2017). On the other hand, the majority of Western research has discovered negative repercussions when parents adopt authoritarian parenting practices (Martínez and García, 2007; Dwairy and Achoui, 2009; Riany et al., 2021). According to the current study, adopting an authoritarian parenting style during the school years may help ensure that children have a strong academic achievement orientation. According to the same study, permissive and authoritative parenting styles are significant predictors of self-efficacy in college students, whereas authoritarian parenting styles are a significant predictor of self-efficacy. The influence of parenting style (authoritative) on academic achievement was determined. The findings suggest a positive impact of parenting style (authoritative) on the students’ academic achievement at the higher secondary school level. The student whose parenting style is control predicts academic achievement among their children at the higher secondary school level. Another findings, Llorca et al. (2017) found no significant relationship between parental influence on subject selection and students’ academic achievement. Arrived at a similar finding, asserting that parental style is a significant predictor of student self-efficacy growth. Similarly, he asserts that authoritative parenting is a key predictor of self-efficacy in the positive direction (Lau, 2013; Wolters and Benzon, 2013; McCardle and Hadwin, 2015; Aldhafri et al., 2020; Khazaie et al., 2021).

Furthermore in the current study Computer use, as measured by frequency of use, improves computer confidence and academic achievement. As a result, the more students who use computers, the more confident they become, and thus the more students who use computers, the better scores in results. Numerous other studies question the established link between computers based LSE and academic achievement (Murillo-Zamorano et al., 2019). According to our findings, the majority of self-regulation components were associated with an academic achievement oriented. These findings corroborate prior findings (Chik and Abdullah, 2018; Lai et al., 2018). To explain this, learners who used more self-regulating mechanisms performed better on future planning and self-efficacy measures. Students with greater cognitive self-regulation can increase their educational achievement by controlling their emotions and emotional influences. Additionally, they are highly motivated to study and are capable of planning properly. There was a significant positive correlation between self-regulation and academic achievement orientation. In other words, the more self-control an individual possesses, the higher his academic performance will be. To clarify this result, one could claim that self-regulation enables an individual to plan for and accomplish multiple future goals. This can be explained by the rapid advancement of computer technology and the extensive use of computers at home, in contrast to the lack of use at school, which is a result of schools’ antiquated equipment and students’ preference for the home environment. Our study also suggests that as computer use increases, parental education enhances academic achievement (Carraher Wolverton et al., 2020), implying that students who use a computer at home do better. Additionally, computer use contributes to a positive classroom environment and academic performance. As a result, despite the fact that computer use at school already has a direct effect on students’ performance, But in Pakistan the majority of students do not have easy access to computers at school (high schools in Pakistan average 4.2 students per computer), but those who do benefit academically (Ameen, 2017; Zhao et al., 2022). According to current research findings, authoritative parenting in children evolves through time as learners engage in instructional circumstances that provide them with information about what effective self-regulation is and when and how it can be implemented. Previous research on the relationship between SRL and achievement has yielded contradictory results, and its application in computer learning contexts appears to be limited, with some authors removing the construct from their SRL evaluation model to improve fit (Vilkova and Shcheglova, 2020). According to the findings, SRL is a critical component of academic performance (Mushtaque et al., 2022d). Students that exhibit a high level of SRL earn a higher GPA. According to Wandler and Imbriale (2017) self-regulation has a beneficial effect on academic achievement. According to Kashif and Shahid (2021) self-regulation is a fundamental component of computer learning and academic achievement. Additionally, the data suggest that all components of self-regulation, including post-learning metacognitive processes, have a significant impact on academic achievement. Moreover, metacognitive activities, time management, and the environment all had a significant effect on academic performance.

The goal of this study was to investigate the factors that influence digital literacy among students of educational levels. Students’ self-reported statistics indicated extremely high levels of use in education. Current study results revealed that students with excellent self-regulated learning, high academic accomplishment, and digital literacy boost their learning achievements and develop their learning attitudes in computer-related courses. This conclusion is congruent with the findings of research. Additionally, the majority of participants in this study reported having moderate to high DL levels. These results could be explained by the fact that all study participants live in a society where technology is used for nearly everything, and as a result, they easily identify as technologically literate. The use of DL contributes to academic success. While some studies discovered exceptional learning outcomes, the majority of learners benefited from technology-assisted instruction (Helsper and Smahel, 2019). Those who received online education without any support from technology developed a negative attitude about it and experienced poor learning outcomes. Despite their capacity to communicate with peers and lecturers, students reported feeling anxious and dissatisfied with this method of online learning. Asniza et al. (2021) stated that advanced technologies have the ability to boost student engagement and provide value to academic activities. Digital literacy has the potential to dramatically improve the process of online learning and teaching. Learners and teachers who are digitally savvy may be more prepared to deal with technical difficulties and online learning issues such as privacy breaches (Hartnett et al., 2011). The use of educational technologies, academic communication, planning of learning activities, assessing learning performance, and sharing information have all benefited from digital literacy. During a pandemic, digital literacy and parental support may be able to help children overcome online learning challenges and optimize the online learning and teaching process (Purnama et al., 2021). Teachers who are more digitally literate and have easy access to online learning materials may be better prepared to deal with an emergency situation. They were willing to adapt their teaching methods to the new conditions of the epidemic, seeing it as a fresh opportunity.

## Conclusion

This study develops a conceptual model for the effect of multiple factors on the academic achievement of high school students. In general, the empirical findings corroborate similar findings in the literature. According to the provided conceptual paradigm, authoritative parenting students have a high SRL and academic achievement. We discovered that children who have a permissive parenting style have low SRL and academic achievement as a result of their usage of computers to “kill time” or play games. These are the students who receive the lowest grades. This conclusion can be drawn from the absence of a correlation between SRL and academic success. Nonetheless, there is a strong correlation between academic advancement and students’ perceptions of computers as teaching tools. Academic attainment is low in students reared by lenient parents. People have less faith in computers as a result of this mindset. A significant finding about digital learning was attained through the moderating effect of SRL and LSE on academic achievement. While digital literacy has a limited association with LSE and academic achievement, SRL improves academic achievement for secondary school students. When children use the computer at home, they must use it frequently in order to have an effect on their academic achievement; but, when kids use the computer in school, the computer has a positive effect on their academic achievement regardless of how frequently they use it. On the other hand, the frequency with which students use computers influences their academic achievement.

### Limitations and future suggestions

The generalizability of the findings is constrained by the study sample’s composition. The current study compiled its conclusions using data from Punjab and public schools. Additional research is required on a broader range of samples within Pakistan, as well as from other parts of South Asia. The limitations of the study were acknowledged. To begin, this was a self-reported study using just student data. In the future, interviews with parents and peers may be undertaken to provide depth to the data. Such variables on academics and self-efficacy can alter the results from those in more amicable conditions. The current study did not examine the effect of socioeconomic status (SES) on academic achievement; rather, income was employed to estimate SES. Generally, obtaining a sample that is more representative of a national sample would result in more descriptive results. According to the results of the Board of Intermediate and Secondary Schools, pupils do poorly in computer-related subjects, and the study’s findings indicate that students lack self-efficacy in computer-based learning. Therefore, it is advised that conferences, seminars, and student training programs be established to teach students about the value of digital literacy and effective learning techniques. The evolution of parenting styles’ impact on self-efficacy and academic achievement would be carried out with a longitudinal design.

## Data availability statement

The raw data supporting the conclusions of this article will be made available by the authors, without undue reservation.

## Ethics statement

The studies involving human participants were reviewed and approved by Department of Education, Nanjing Normal University, China. Written informed consent to participate in this study was provided by the participants’ legal guardian/next of kin.

## Author contributions

All authors listed have made a substantial, direct, and intellectual contribution to the work and approved it for publication.

## Funding

The Corresponding Author received the funding from the International Joint Research Project of Huiyan International College, Faculty of Education, Beijing Normal University, China (ICER202001).

## Conflict of interest

The authors declare that the research was conducted in the absence of any commercial or financial relationships that could be construed as a potential conflict of interest.

## Publisher’s note

All claims expressed in this article are solely those of the authors and do not necessarily represent those of their affiliated organizations, or those of the publisher, the editors and the reviewers. Any product that may be evaluated in this article, or claim that may be made by its manufacturer, is not guaranteed or endorsed by the publisher.
